# Harmonizing minds and machines: survey on transformative power of machine learning in music

**DOI:** 10.3389/fnbot.2023.1267561

**Published:** 2023-11-10

**Authors:** Jing Liang

**Affiliations:** Department of Music, Zhumadian Preschool Education College, Henan, China

**Keywords:** machine learning, automatic music, music recommendation, music classification, music generation, music transcription, applications

## Abstract

This survey explores the symbiotic relationship between Machine Learning (ML) and music, focusing on the transformative role of Artificial Intelligence (AI) in the musical sphere. Beginning with a historical contextualization of the intertwined trajectories of music and technology, the paper discusses the progressive use of ML in music analysis and creation. Emphasis is placed on present applications and future potential. A detailed examination of music information retrieval, automatic music transcription, music recommendation, and algorithmic composition presents state-of-the-art algorithms and their respective functionalities. The paper underscores recent advancements, including ML-assisted music production and emotion-driven music generation. The survey concludes with a prospective contemplation of future directions of ML within music, highlighting the ongoing growth, novel applications, and anticipation of deeper integration of ML across musical domains. This comprehensive study asserts the profound potential of ML to revolutionize the musical landscape and encourages further exploration and advancement in this emerging interdisciplinary field.

## 1. Introduction

Music, a universal language transcending cultural and linguistic barriers, has always been fertile ground for the advent and progression of technology. From the first simple mechanical devices that created sound to the sophisticated digital platforms enabling global music streaming, technology has continuously reshaped the music landscape (Cross, 2001). The most recent and promising addition to this technological symphony is machine learning, a revolutionary field within artificial intelligence. Machine Learning (ML), characterized by its ability to learn and improve from experience without explicit programming (Zhou, 2021), is infiltrating various domains, pushing the boundaries of innovation and traditional paradigms. A harmonious and transformative synergy is born when it encounters the expansive realm of music. As it ventures into music information retrieval, automatic music transcription, music recommendation, and algorithmic composition, ML leaves an indelible footprint on the musical world (Briot et al., 2020). Furthermore, the authors (Safron, 2020) proposed using the Free Energy Principle and Active Inference Framework (FEP-AI) to integrate leading theories of consciousness with ML approaches to modeling the brain. The authors argue that this integration can help bridge the gap between cognitive science and AI research and lead to a more comprehensive understanding of how the brain generates conscious experience.

The impact of music depends on several factors, including cultural and individual differences, as human cognition is a complex and multifaceted phenomenon (Juslin et al., 2022). Therefore, any ML interpretations of cognitive harmony through music must be considered in the context of these complexities. Additionally, the notion of cognitive harmony raises questions about the universality of musical impact across different cultures and societies (Athanasopoulos et al., 2021). How do cultural backgrounds and individual experiences shape our cognitive responses to music? ML can help explore these questions by analyzing large datasets of music preferences and cognitive responses across diverse populations (Modran et al., 2023). The effectiveness of different technologies in musical ML hinges on various factors. Deep learning neural networks excel at handling complex, high-dimensional musical data, making them ideal for tasks like music classification (Nasrullah and Zhao, 2019). In contrast, traditional ML methods are more efficient and interpretable, making them suitable for more straightforward tasks or resource-constrained environments (Ali et al., 2023). The choice depends on the specific musical task, data availability (Gelding et al., 2019), computational resources (Goltz and Sadakata, 2021), interpretability needs (Afchar et al., 2022), domain knowledge integration, and the level of personalization required by end-users (Afchar et al., 2022). Thus, selecting the right technology in musical ML involves a nuanced assessment of these factors to optimize performance in different applications.

Music has been a significant part of human culture and continues to play an essential role in our lives. However, with the advent of technology and the growth of ML, music is no longer limited to human performance and composition. ML can transform how we create, listen to, and understand music. Everyday living includes music in a significant way. The familiar music system works to arouse our sense of hearing through music, allowing us to experience the feelings expressed in music (Cella, 2020). Through this feeling, we can sense the music and unique emotions in the social setting. When considering music to be an abstract art, with synaesthesia, hearing and visual are similar in their fundamental and psychological characteristics (Curwen, 2022); therefore, they frequently work in concert to produce superior outcomes. Other than the most direct hearing, current psychology states that combining several sensory systems creates the cognition of music (Ilari, 2021). The ear, an auditory pleasure, completes music according to the conventional notion. However, people have now recognized that when we listen to music, we frequently experience picture-thinking processes like emotion and image association without realizing it. As a result, when listening to music, we might use our imagination to gather information about the music's image and creative idea and see it as an inevitable trend. For instance, music and visual scenes have made for some of the best pairings in art in the past.

Music recommendation, classification, transcription, and generation are vital areas where ML has been applied. In music recommendation, ML algorithms have been utilized to predict the songs a user will likely listen to base on their listening history (Paul and Kundu, 2020). Music classification automatically categorizes music into various genres, and ML algorithms have been used to improve the accuracy of music classification (Chillara et al., 2019). Music transcription refers to converting audio signals into musical notation, and ML algorithms have been used to improve the accuracy and efficiency of music transcription (McVicar et al., 2014). ML algorithms have been utilized in music generation to generate new music based on inputs such as existing music pieces, lyrics, or emotions (Zhao et al., 2022). The authors (Tang et al., 2022) focused on applying deep learning (DL) and ML techniques in music education. The authors (Novelli and Proksch, 2022) explored the limitations of current music-generating AIs regarding emotional awareness and proposed potential solutions to address this issue, discussing the potential impact of emotionally-aware music-generating AIs on the music industry.

Despite the progress made in ML-empowered music, challenges and limitations still need to be addressed. For example, generating music with machine-learning models indistinguishable from human-composed music is still an ongoing challenge (Ji et al., 2020). Similarly, music recommendation systems still face challenges such as data sparsity and scalability (Goyani and Chaurasiya, 2020). Additionally, ML models have been used to recognize emotions in music (He, 2022). The authors (Charitou, 2023) highlighted the various approaches used for music synthesis, including ML models, recurrent neural networks, and Generative Adversarial Networks (GAN). In Benetos et al. (2018) introduced the concept of music transcription and its importance, followed by a discussion of the various approaches proposed for music transcription. Omowonuola et al. (2022) focused on developing a music recommendation system that considers the music's context and content.

Music is a profound and universal form of human expression that transcends geographic and cultural boundaries (Patel, 2010). Digital technology has revolutionized how we create, distribute, and consume music (Cook et al., 2019). Simultaneously, ML, a subset of artificial intelligence, has emerged as a powerful tool for data analysis and prediction (Jordan and Mitchell, 2015). These parallel developments have converged in the Music Information Retrieval (MIR) field, a multidisciplinary research domain focusing on extracting information and knowledge from music (Downie, 2003). Our comprehensive study explores the fascinating interaction between ML and music. This intersection has birthed innovative applications like music recommendation systems, classification, transcription, and generation. Each area has evolved dramatically, from basic rule-based systems to sophisticated machine-learning models that can learn from and generate complex musical structures (Sturm, 2014; Ricci et al., 2015; Briot et al., 2017; Benetos et al., 2018). This paper surveys the developments, providing a detailed analysis of current methodologies and highlighting the potential for future innovation. We aim to provide a comprehensive understanding of how ML has been and can be used to augment our interaction with music.

### 1.1. Motivation and contributions

In the epoch of rapid technological advancement, music, one of the most profound expressions of the human spirit, has not been left untouched. The bond between music and technology has always been intimate, evolving from simple instruments to complex digital platforms that shape our musical experiences today (Cross, 2001). However, we stand on the precipice of a new age defined by ML and music convergence. This powerful fusion is not merely reshaping how we interact with music but also redefining the boundaries of creative expression. ML, an offshoot of artificial intelligence, is a beautiful tool increasingly employed in diverse fields, from healthcare and finance to the social sciences. ML's ability to learn from data and make intelligent predictions and decisions have transformed our world, but its integration with music is still in its infancy. Today, as ML algorithms tread the hallowed grounds of music creation, analysis, and consumption, we ask what harmonious melodies this union can bring forth. Can the mathematical rigidity of algorithms capture the emotional fluidity of music? This study's motivation is rooted in exploring and understanding these compelling questions. It seeks to delve into the intersections of ML and music, not just as isolated disciplines but as a harmonious blend of art and science. This research explores the untapped potential of ML in music and how it might improve musical accessibility, discovery, and creativity.

Moreover, the urgency to undertake this comprehensive study stems from the ethical considerations of ML and music. Issues surrounding authorship, copyright, and the potential displacement of human musicians by AI systems are of paramount importance (Miranda, 2021). The study addresses these concerns while charting future research and development paths while balancing technological innovation and ethical responsibility. Indeed, the harmony of ML and music forms a fascinating symphony, rich in complexity and full of possibilities. This survey, therefore, presents a deep dive into this confluence, driven by the quest to understand, explore, and ultimately contribute to the future evolution of music in the era of ML. The survey contributions summary is as follows.

- We provide a comprehensive survey of the application of ML in music, covering key areas like music recommendation, music classification, music transcription, and music generation.
- We analyze the evolution of methodologies in these areas, from basic rule-based systems to sophisticated ML models. This historical perspective allows a better understanding of the current state of the art and potential future directions.
- We discuss this field's significant difficulties, such as model interpretability, data handling, and the elusive objective of achieving true creativity in machine-generated music.
- We introduce this field's significant difficulties, such as model interpretability, data handling, and the elusive objective of achieving true creativity in machine-generated music. Furthermore, we identify opportunities for future research and innovation in ML and mu- sic, offering insights into promising areas for further exploration.

### 1.2. Related surveys

The authors (Chillara et al., 2019) provided a comprehensive overview of the current state-of-the-art music genre classification using ML algorithms. The survey focused on the different approaches and techniques used for music genre classification and evaluated their performance. In Harshvardhan et al. (2020) explored the use of ML for music information retrieval. This survey discussed the various tasks in music information retrieval and the corresponding ML algorithms used for each task, including music genre classification, similarity analysis, and music recommendation. Also, Zhang et al. (2019) focused on DL applications in music information retrieval. The survey discussed using DL models for music genre classification, tag prediction, and recommendation tasks. The authors also discussed the challenges and limitations of DL models in the music domain and summarized the current state-of-the-art for each task. In Ji et al. (2020), conducted a comprehensive survey on music generation and discussed the various machine-learning algorithms used for music generation. The authors also discussed the challenges and limitations in the field and highlighted the recent advancements in music generation.

One intriguing perspective involves considering whether music's profound impact on human minds is due to an inherent cognitive harmony (Peretz and Zatorre, 2005). In this context, ML, particularly advanced techniques like neural networks, can be applied to analyze music's structural and emotional components and how they align with human cognition (McDermott and Simoncelli, 2011). ML algorithms can dissect the structural elements of music, including rhythm, melody, harmony, and timbre (Choi et al., 2017a). By comparing these elements with patterns in brain activity, researchers can explore whether music resonates with innate cognitive structures. ML models can also be employed to analyze emotional responses to music (Song et al., 2016). By examining physiological data such as brain activity (Schaefer, 2017). At the same time, individuals listen to music, it is possible to uncover patterns that suggest a deep connection between musical harmonies and emotional states. ML can be used to build predictive models that anticipate how a piece of music will affect an individual's mood or cognitive state based on its inherent characteristics (Abdul et al., 2018). The approach can show whether there are consistent cognitive responses to certain musical features. The authors (Safron, 2020) Integrated World Modeling Theory of Consciousness combines various theories and frameworks to provide a comprehensive account of how the brain generates conscious experience, used ML approaches to model the brain and study the neural correlates of consciousness to develop more accurate and sophisticated models of the brain and its functions.

The authors (Harshvardhan et al., 2020) focused on applying generative models to ML, including their use in music generation. The survey provided an overview of the different types of generative models, including GANs, variational autoencoders (VAEs), and recurrent neural networks (RNNs). In addition, it highlighted each model's strengths and limitations and discussed the most recent research and advancements in the field. The authors (Briot et al., 2017) highlighted the state-of-the-art models, algorithms, and methods used for music generation and their applications in different domains. Additionally, it compared other DL techniques and their limitations. The authors (Ji et al., 2020) provided a thorough overview of the profound music generation's current state of the art. Furthermore, the authors thoroughly analyzed the various DL techniques used for music generation and the challenges faced in this field.

These surveys demonstrate the importance and relevance of ML-empowered music and highlight the advances made in different areas of this field. The present survey aims to build on these surveys by providing a comprehensive overview of state of the art in music recommendation, classification, transcription, and generation and highlighting the challenges and limitations that still need to be addressed in each area. Table 1 describes the most related surveys in our survey domain.

**Table 1:** Summary of the existing surveys.

**References**	**Highlighted**	**Focused**	**Limitation**	**Future directions**
Chillara et al. (2019)	Using a decision tree, KNN, and SVM for music genre classification	The performance	Single evaluation metric	Evaluating the performance using multiple metrics
Harshvardhan et al. (2020)	Using VAEs, CNN, and RNNs for generative models	Applications of generative models	Comparison of generative models for music	Generative models for music applications
Zhang et al. (2019)	Using DNNs, RNN, and CNN for music recommendation	Challenges and opportunities of DL-based recommender systems	Comparison of DL-based recommended systems for music	Exploring the applications in different domains
Ji et al. (2020)	Using DNNs, RNNs, and CNN for deep music generation	Multi-level representations and evaluations	Evaluation of deep music generation	Comparison of deep music generation algorithms
Briot et al. (2017)	Using DNNs and RNNs for music generation	Applications of DL for music generation	Evaluation of DL techniques for music generation	Music generation applications
Ji et al. (2020)	Using DL models for improved music generation	DL techniques for music generation	More diverse training data	Better evaluation metrics.

### 1.3. Survey structure

The rest of the survey paper is organized as follows. Section 2 presents music transportation, and music recommendation is presented in Section 3. Section 4 discusses music classification, while music generation is introduced in Section 5. Finally, the paper is concluded in Section 6.

## 2. Music transcription

ML for music transcription is a rapidly growing field that has the potential to revolutionize the way we interact with and understand music. The goal of music transcription is to convert audio recordings of music into a symbolic representation, such as sheet music or MIDI files. This process is typically done manually, but with the advent of ML techniques, it has become possible to automate this task. This paper will discuss the current state of the art in ML for music transcription, including its applications, challenges, and future directions.

Music transcription is converting audio recordings of music into a symbolic representation, such as sheet music or MIDI files. Music transcription converts audio signals into a symbolic representation of the music, such as sheet music or MIDI files. It is an important problem in music information retrieval (MIR) because it allows the analysis and manipulation of music in a symbolic form. There are several types of music transcription, including monophonic, polyphonic, and multimodal transcription. Monophonic transcription involves converting a single instrument audio signal into sheet music, while polyphonic transcription involves transcribing multiple instruments playing simultaneously. The ability to transcribe music automatically has many potential applications, such as music education, music analysis, and music search. This paper will discuss the current state of the art in ML for music transcription, including its applications, challenges, and future directions. ML techniques have been widely used in music transcription, which aims to convert audio recordings of music into symbolic representations, such as MIDI or sheet music. ML for music transcription is an active area of research that aims to use computational methods to automatically transcribe audio recordings of music into symbolic representations such as sheet music or MIDI files. This task is challenging due to the complexity of the audio signal, the variability of musical styles, and the subjectivity of musical notation. ML has been widely used in music transcription, which converts audio signals into symbolic representations such as sheet music or MIDI. The application of ML in music transcription is multi-faceted and includes tasks such as pitch detection, onset detection, and chord estimation.

One of the main applications of ML for music transcription is music education. Automatic transcription can help students learn to read music by providing a way to view a song recording in a form they can understand. Additionally, automatic transcription can create sheet music or MIDI files for songs without existing notation. Another application of ML for music transcription is music analysis. Automatic transcription can extract information about a piece of music's structure, harmony, and melody, which can be used to study the style and influences of different composers and performers. Music transcription has several essential applications in the field of MIR. For example, it can generate sheet music from audio recordings, which can be used for educational and creative purposes. It can also be used to create MIDI files from audio recordings, which can be used to control synthesizers and other musical instruments. Additionally, music transcription can improve the search and retrieval of music in large databases.

Music transcription is vital because it converts audio recordings of music into symbolic, notated forms. This allows for a more precise and accurate representation of a piece of music, enabling further analysis, manipulation, and understanding of the music. In addition, music transcription can be used to digitize older recordings and make them accessible to a wider audience. Additionally, music transcription can be used to produce sheet music for educational and performance purposes, making it easier for musicians to learn and play new pieces of music. Music transcription is crucial as it enables a deeper understanding and preservation of music, making it accessible and usable for various purposes.

### 2.1. Background

The traditional approach to music transcription is based on applying signal processing techniques, such as pitch detection, onset detection, and rhythm estimation. These techniques extract features from the audio signal, which are then used as input to a transcriber. However, these methods have several limitations, such as the difficulty of dealing with polyphonic music and the need for manual parameter tuning. In recent years, ML techniques have been successfully applied to the problem of music transcription, making it possible to transcribe music automatically and at high accuracy. ML for music transcription can be divided into two main categories: audioto-score and audio-to-MIDI. Audio-to-score transcription involves converting audio recordings of music into sheet music, which can include notes, chords, and other symbols. Audio-to-MIDI transcription involves converting audio recordings of music into MIDI files, which can consist of information about the letters, timing, and other performance attributes.

Researchers have also explored the use of ML techniques such as support vector machines (SVMs) and hidden Markov models (HMMs) for music transcription (Yegnanarayana and Murty, 2009; Benetos and Dixon, 2013). Other approaches include using SVMs (Lu et al., 2005), which have been used to transcribe monophonic audio and have been shown to achieve comparable results to DNNs. Recent advances in ML have led to significant improvements in the accuracy and efficiency of music transcription systems. One key approach is using DNNs, which are effective for tasks such as pitch detection, onset detection, and rhythm estimation (Liu et al., 2010; Deng and Kwok, 2016). Another critical area of research is RNNs for music transcription. RNNs are particularly well-suited for this task because they can process sequential data and effectively model the temporal structure of music (Choi et al., 2017b; Hadjeres et al., 2017). Early research in this area focused on using rule-based methods and HMM (Benetos and Dixon, 2013). However, with the advent of DL techniques, such as RNNs and CNNs, the performance of music transcription systems have significantly improved (Boulanger-Lewandowski et al., 2012; Schneider et al., 2021).

Alfaro-Contreras et al. (2023) proposed a fusion approach where image and audio information are processed separately and combined in a late stage for music transcription. Also, (Reddychakradhar Goud et al., 2022) discussed various music transcription techniques and how they can be connected to achieve real-time results. In George et al. (2022) proposed a hybrid model that combines different ML algorithms, including k-nearest neighbor (KNN), SVM, Random Forest (RF), and artificial neural networks to achieve better performance. The authors (Huaysrijan and Pongpinigpinyo, 2022) focused on developing an ML-based system for automatically transcribing music played on a Thai xylophone with soft mallets.

One of the most popular approaches for music transcription is using deep neural networks (DNNs) (Rom a'n et al., 2020). DNNs have been used to transcribe a variety of music signals, including monophonic and polyphonic audio, and have shown promising results in transcription accuracy. Another approach is using CNNs (Sleep, 2017; Schneider et al., 2021), which have been used to transcribe monophonic audio and have been shown to achieve comparable results to DNNs. Another approach is using RNNs (Wu et al., 2020), which have been used to transcribe polyphonic audio and have been shown to achieve comparable results to DNNs. In addition to traditional audio-to-symbolic transcription, recent research has also focused on developing end-to-end models that transcribe audio recordings to sheet music (Meng and Chen, 2020). These models have shown promising results and have the potential to revolutionize the field of music transcription. Another important area of research is the use of RNNs for music transcription. RNNs are particularly well-suited for this task because they can process sequential data and effectively model the temporal structure of music (Hadjeres et al., 2017; Chen et al., 2019).

### 2.2. Challenges

One of the main challenges in ML for music transcription is dealing with polyphonic music. Polyphonic music is music that has multiple parts playing at the same time, which can make it difficult to transcribe accurately. MLfor music transcription is also still in its infancy, and many open research questions must be addressed. Despite the recent advances in ML for music transcription, several challenges remain to be addressed. One of the main challenges is dealing with polyphonic and non-piano music, as traditional transcription methods were developed for monophonic piano music. Another challenge is the lack of a large amount of labeled data needed to train ML models. Additionally, there are issues with dealing with tempo and pitch variations and incorporating additional modalities, such as lyrics or body movements, in multimodal transcription.

One of the main challenges in music transcription is dealing with polyphonic music, where multiple notes are played simultaneously. Researchers have proposed various methods to address this challenge, such as integrating attention mechanisms in RNNs (Wu et al., 2021). Another challenge is dealing with monophonic music, where only one note is played. To address this challenge, researchers have proposed the use of pitch detection algorithms (Schedl et al., 2014; Burgoyne et al., 2015) and the integration of pitch estimation algorithms in CNNs (Meng and Chen, 2020). Despite these advances, many challenges still need to be addressed to improve the accuracy and robustness of music transcription systems. These include dealing with variations in performance style, handling multiple instruments and polyphonic music, and dealing with the subjectivity of musical notation.

### 2.3. Future directions

In the future, ML for music transcription will likely improve and become more accurate. Additionally, it is possible that new techniques will be developed that can transcribe a wider range of music, including polyphonic music and music with complex rhythms. There are several promising future directions for research in ML for music transcription. One of the main directions is the development of models that can handle polyphonic and non-piano music. Another direction is the use of unsupervised and semi-supervised learning techniques, which can enhance the effectiveness of machine learning models.

#### 2.3.1. Summary

ML has played a crucial role in developing music transcription systems. With the continued advancement of DL techniques, we can expect to see even more accurate and efficient systems. However, challenges remain to be addressed, such as dealing with polyphonic music and developing models that can transcribe audio recordings to sheet music. ML is an essential tool for music transcription, enabling the automatic transcribing of music audio recordings into symbolic representations. The recent advances in DL have led to significant improvements in the accuracy and efficiency of music transcription systems. However, many challenges still need to be addressed to improve the performance of music transcription systems further. Table 2 illustrates the advantages and disadvantages of ML techniques for better performance in music transcription.

**Table 2:** Comparison of different ML techniques for music transcription.

**Technique**	**Advantages**	**Disadvantages**
DL	Handle large amounts of data, better representation of complex musical features	Requires a large amount of labeled data and computational resources
Hidden Markov Models (HMM)	Good at modeling sequential patterns, can handle large amounts of data	Difficulty in modeling the complex structure of music
SVM	Handle high-dimensional data and work well with limited data	Prone to overfitting with large amounts of data and complexity in modeling musical features
Dynamic Time Warping (DTW)	Effective in handling time series data and flexible in matching musical phrases	Prone to errors in musical alignment and limited representation of musical features
KNN	Simple and flexible can handle multi-dimensional data	Poor performance with highdimensional data and large amounts of data

## 3. Music recommendation

Music recommendation systems have become increasingly popular as they allow users to discover new music tailored to their tastes. ML techniques have played a crucial role in developing these systems, enabling them to learn from large amounts of data and make accurate predictions about which songs a user is likely to enjoy. In this research paper, we will explore the use of ML for music recommendation, including the different techniques used, the challenges encountered, and the future directions for research in this area.

Music recommendation systems are designed to help users discover new music similar to the music they already like. They have become increasingly popular in recent years, as streaming services such as Spotify and Apple Music have made accessing large music libraries easier. The success of these systems depends on their ability to predict which songs a user is likely to enjoy accurately, and ML techniques have played a crucial role in achieving this.

Music recommendation is important because it helps individuals discover new music that aligns with their tastes and preferences. With the vast amount of music available today, music recommendation systems provide a solution for filtering out irrelevant music and suggesting only the most relevant pieces to the individual. This not only saves time for the listener but also helps to promote new music and artists. Furthermore, music recommendation systems can also provide insights into music trends and audience preferences, which can be helpful for music producers and marketers.

There are several ways in which ML has been used in music recommendation systems. One of the most common approaches is to use collaborative filtering, which involves learning from the listening habits of a group of users. This can be done by analyzing users' listening history and identifying patterns in the songs they have listened to. Another approach is to use content-based filtering, which involves learning from the characteristics of the music itself. This can be done by analyzing the audio features of songs, such as the tempo, melody, and harmony.

### 3.1. Background

ML is a branch of artificial intelligence that uses algorithms to learn from data and make predictions. It has been used in various applications, including image and speech recognition, natural language processing, and, more recently, music recommendation. ML use in music recommendation systems has been motivated by a large amount of available data, including information about the music (e.g., genre, tempo, and lyrics) and the listeners (e.g., demographics, listening history, and playlists). Music recommendation includes collaborative filtering, matrix factorization, and DL methods. Collaborative filtering, such as user-based and item-based methods, utilizes past user interactions with items to make recommendations. Matrix factorization techniques, such as singular value decomposition and non-negative matrix factorization, factorize the interaction matrix of users and items into latent representations for users and objects. These latent representations can then be used for making recommendations.

DL methods have also been applied to music recommendation, such as using multi-layer perceptrons (MLP) and RNN. These methods effectively capture the complex patterns in the user-item interactions and have achieved state-of-the-art performance on music recommendation benchmarks (Lin et al., 2019; Schedl, 2019; Dang et al., 2021; Wang et al., 2021; Singh et al., 2022). In addition, Schindler et al. (2012) proposed to use metadata information from songs to improve the accuracy of music recommendations and Kim et al. (2020) used DL for music recommendation by using user listening history and audio features of songs as input. In Rendle (2010) proposed a factorization machine model for music recommendation, which is a generalization of matrix factorization and is effective in recommendation tasks.

Furthermore, the authors Elbir and Aydin (2020) presented a DL approach for music recommendation, using a combination of CNN and long short-term memory (LSTM) to model a user's listening history and music characteristics. In Pretet et al. (2022) presented an extension of the method in the first reference, using a deep neural network to model the music content and the user's listening history. Moreover, Elbir and Aydin (2020) presented a method for music genre classification using deep convolutional neural networks. Also, the authors Lops et al. (2019) presented a content-based music recommendation system that uses tag-based similarity and semantic relationships between songs to recommend similar music to the users. In Chheda et al. (2023) proposed a new music recommendation approach based on the emotional content of associated images. Also, La Gatta et al. (2022) proposed a music recommendation system that utilizes hypergraph embedding for feature representation and similarity measurement. The authors (Velankar and Kulkarni, 2022) provided a comprehensive overview of the current state of music recommendation systems and the challenges.

### 3.2. Challenges

Despite the success of ML in music recommendation, several challenges still need to be addressed. One of the main challenges is the cold start problem, which occurs when a system cannot make recommendations for new users who have not yet provided any listening history. Another challenge is the system's scalability as the number of users and songs grows. Finally, there is the issue of diversity in recommendations, which is essential to ensure that users are exposed to various music styles and genres.

### 3.3. Future directions

The ML field for music recommendation is still relatively new, and there is much room for further research. One of the main research areas is using DL, a type of ML involving neural networks. Another area of research is the integration of other forms of data, such as social media and lyrics, to make more personalized recommendations.

#### 3.3.1. Summary

ML has played a crucial role in developing music recommendation systems, enabling them to learn from large amounts of data and make accurate predictions about which songs a user will likely enjoy. However, several challenges, such as the cold start problem and the system's scalability, still need to be addressed. Future research in this area will likely focus on using DL and integrating other forms of data to make more personalized recommendations. Table 3 shows technique strengths and weaknesses specific requirements.

**Table 3:** Comparison of different ML techniques for music recommendation.

**Technique**	**Advantages**	**Disadvantages**
Collaborative filtering	Personalized recommendations based on user behavior	Struggles with data sparsity and cold start problem
Content-based filtering	Recommendations based on specific features of the music	Does not take into account the preferences of other users
Hybrid recommendation	Combines the strengths of both Collaborative and Content-Based Filtering	More complex to implement
DL	Learn complex patterns in the data	Requires large amounts of data to train
Matrix factorization	Handle large scale data efficiently	Computationally expensive
Association rule mining	Providing additional insights into the relationships between items	Limited by data sparsity

## 4. Music classification

Music classification is the task of automatically categorizing music into different genres, styles, and moods. ML techniques have been widely used to solve this task and have shown to be effective in accurately classifying music. This paper aims to provide an overview of the current state-of-the-art music classification using ML, including feature-based classification, DL, and ensemble methods. Additionally, we will discuss the challenges and future directions in music classification research.

Music classification is a task that has been widely studied in music information retrieval (MIR). This task is essential for music organization, tagging, and retrieval. With the increasing amount of music available online, there is a growing need for music classification methods to automatically categorize music into different genres, styles, and moods. ML techniques have been widely used to solve this task and have shown to be effective in accurately classifying music.

Music classification is vital for many reasons. Firstly, it helps organize extensive music collections by grouping similar musical pieces together. This makes searching for and finding specific music details easier, particularly useful for large music libraries. Additionally, music classification is used to identify the genre of a piece of music, which can be useful in recommending music to users based on their preferences. Music classification can also be used to analyze and study musical trends and the relationships between different types of music. This can be useful for music researchers, musicologists, music educators, and individuals and organizations involved in the music industry. Moreover, music classification can train ML algorithms for music-related applications, such as music recommendation, transcription, and generation. In these applications, music classification can provide the necessary data for the ML algorithms to learn from, allowing the algorithms to make better predictions and generate more accurate results.

### 4.1. Background

ML for music classification is an active area of research, with a growing number of studies applying various ML techniques to classify music into different genres, moods, or other categories. One of the earliest studies in music classification was the work by Tzanetakis and Cook (2002), who used a combination of timbre, pitch, and rhythm features to classify music into 10 different genres. They used a support vector machine (SVM) classifier and achieved an overall accuracy of 85%. In Cheng et al. (2020) proposed a deep convolutional neural network (CNN) model for music genre classification. They used a dataset of 1 million songs and achieved an overall accuracy of 85.4%, which significantly improved over the previous state-of-the-art results. In addition, the authors (Choi et al., 2017b) proposed a method for music classification using a combination of CNN and recurrent neural network (RNN) models. The achieved an overall accuracy of 90.2% on a dataset of 10 genres, demonstrating the proposed method's effectiveness. Li et al. (2022) proposed a method for music classification using a transformer-based neural network architecture. The achieved an overall accuracy of 92.6% on a dataset of 10 genres, showing the effectiveness of transformer-based models for music classification.

The authors (Bahuleyan, 2018) proposed a method for music genre classification using ML techniques. They used a dataset of 10 genres and achieved an accuracy of 96.42%. This paper contributes to the field of music genre classification by comparing the performance of various ML techniques for this task. In Morfi and Stowell (2018) introduced a DL method for audio event recognition and active detection. It focused on the challenges and future directions in this field. This paper contributes to the field of music classification by highlighting the use of DL for audio event recognition and active detection. While Shah et al. (2022) presented at International Conference on Control, Automation and Systems in 2017, proposed a method for music genre classification using deep convolutional neural networks. They used a dataset of 10 genres and achieved an accuracy of 92.5%. This paper contributes to the field of music genre classification by demonstrating the effectiveness of DCNNs in this task.

Furthermore, the authors (Tzanetakis and Cook, 2002) reviewed various techniques used for music genre classification. They discussed the advantages and disadvantages of each method. This paper contributes to the field of music genre classification by providing a comprehensive overview of the existing techniques. Also, in Kumaraswamy and Poonacha (2021) proposed a method for music genre classification using deep convolutional neural networks. They used a dataset of 10 genres and achieved an accuracy of 97.2%. This paper contributes to the field of music genre classification by demonstrating the effectiveness of DCNNs in this task. In Choi et al. (2017b) proposed a method for music classification using deep RNNs. He discussed the challenges and future directions in this field. This paper contributes to the field of music classification by highlighting the use of deep RNNs for this task. Moreover, the authors (Shah et al., 2022) proposed a method for music genre classification using deep CNNs. They used a dataset of 10 genres and achieved an accuracy of 91.3%. This paper contributes to the field of music genre classification by demonstrating the effectiveness of DCNNs in this task. The authors (Khan et al., 2022) explored the impact of feature selection on the accuracy of music popularity classification using ML algorithms. In Wu (2022) focused on using ML algorithms to classify emotions in ethnic music, such as happiness, sadness, anger, and others, based on features extracted from the music. Furthermore, Ashraf et al. (2023) presented a hybrid model for music classification using a combination of Convolutional Neural Network (CNN) and Recurrent Neural Network (RNN) variants to improve the accuracy of music classification.

In summary, the literature in this area has demonstrated that ML techniques, such as SVMs, CNNs, RNNs, and transformers, can be effectively applied to music classification tasks. However, many challenges remain to be addressed, such as dealing with small and imbalanced datasets and improving the interpretability of the models.

### 4.2. Challenges

The main challenges of music classification are the feature space's high dimensionality, the music signal's variability and the lack of large labeled music datasets. Additionally, the lack of standardization of music genres, styles and moods can also challenge music classification.

### 4.3. Future directions

In the future, music classification research will focus on developing methods that can handle the feature space's high dimensionality and the music signal's variability. Additionally, the study will focus on developing methods that can handle music with multiple genres, styles, and moods. Furthermore, the research will focus on methods to handle music with different languages, cultures, and regions.

#### 4.3.1. Summary

Music classification is an essential task in the field of music information retrieval (MIR). ML techniques have been widely used to solve this task and have shown to be effective in accurately classifying music. This paper provided an overview of the current state-of-theart in music classification using ML, including feature-based classification, DL, and ensemble methods. Additionally, we discussed the challenges and future directions in music classification research. Table 4 shows the strength and weaknesses of different ML techniques used for music classification. It is important to note that the choice of technique will depend on the specific requirements of the task and the available resources.

**Table 4:** Comparison of different ML techniques for music classification.

**Technique**	**Advantages**	**Disadvantages**
Decision Tree	Easy to understand and interpret handle both categorical and numerical data	Overfitting, prone to instability when small variations in the data occur.
RF	Better performance than a single decision tree, reduces overfitting, can handle large datasets.	Computationally expensive, may still overfit when there are many trees.
Naive Bayes	Fast and efficient, easy to implement, well suited for highdimensional datasets.	Assumes independence between features, which may not always be accurate.
SVM	Handle non-linear data, good for high-dimensional datasets, efficient for large datasets.	Choosing the correct kernel can be difficult, and may not perform well for noisy data.
CNN	Performance for highdimensional data, flexible to model different types of audio signals	Requires large amounts of labeled data, can be difficult to interpret
RNN	Good for modeling temporal dependencies in audio signals can handle variable-length input	Difficult to train, and requires large amounts of labeled data.

## 5. Music generation

Music generation is the task of creating new music using computational methods. ML techniques have been successfully applied to this task, allowing for the creation of unique and diverse music. Music generation using ML is a rapidly growing field that has the potential to revolutionize the music industry. The use of ML algorithms allows for the creation of new and unique pieces of music and the ability to mimic the style of a particular artist or genre. This section will explore the various techniques used in music generation and their corresponding applications, challenges and future directions.

Music generation is essential as it provides a new way to create musical pieces through artificial intelligence. This opens up opportunities for artists, composers, and musicians and allows for creation of new musical styles and forms. The music generation process involves feeding large amounts of musical data into ML algorithms and generating new pieces based on that data. This process can create unique and diverse music for various applications, such as film scores, video game soundtracks, and more. In addition, music generation can also be used for research purposes, such as studying the structure and evolution of musical styles and forms and exploring artificial intelligence's creative potential. With the growing interest and development in ML, music generation is expected to become an increasingly important area of research and application. The applications of ML for music generation are numerous, including the age of melodies, rhythms, harmonies, and even entire songs. These techniques can be used in various domains, such as music production, education, and entertainment. For example, in music production, ML can be used to generate new melodies or chord progressions. In contrast, in music education, it can be used to teach students about music theory and composition. Additionally, ML techniques can be used to create personalized music for video games, films, and other forms of media. Music generation using ML has a wide range of applications, including:

## 6. Music composition

ML algorithms can be used to generate new pieces of music, either by mimicking the style of a particular artist or by creating something entirely new. Music composition using ML involves using algorithms and models to generate new musical content. This can be done through Markov models, neural networks, and evolutionary algorithms. These techniques can create melodies, harmonies, and rhythms and control various aspects of music, such as style, tempo, and timbre.

One of the key challenges in music composition using ML is creating algorithms that can generate musically coherent and high-quality music. This requires a deep understanding of music theory and the ability to model complex musical relationships. Additionally, there is a need for large amounts of training data and powerful computing resources. There are a number of different approaches to music composition using ML, and the field is still an active area of research. Some examples of recent work in this area include using DL to generate music that mimics the style of a particular composer (Liu and Ramakrishnan, 2014), using RL to compose music in a specific style (Sulun et al., 2022), and using evolutionary algorithms to generate new melodies (Marques et al., 2000).

Overall, ML has the potential to revolutionize the field of music composition by enabling the creation of new and unique musical content. However, many challenges still need to be overcome, and further research is needed to realize this potential fully.

## 7. Music arrangement

ML can be used to rearrange existing pieces of music, creating new versions of the same piece. The music arrangement is the process of organizing the various elements of a piece of music, such as melody, harmony, rhythm, and timbre, to create a cohesive and pleasing final product. ML, on the other hand, is a subfield of artificial intelligence that involves training algorithms to learn patterns and make predictions based on data. Combining music arrangement and ML can lead to many exciting possibilities, such as the automatic generation of new and unique arrangements, the ability to personalize music for individual listeners, and the automation of tedious and repetitive tasks. One example of music arrangement using ML is the work of researchers at the Georgia Institute of Technology (Ali and Siddiqui, 2017). They developed a system that uses ML to analyze a given piece of music and generate new arrangements by adjusting parameters such as instrumentation and tempo. This system created arrangements that were both musically interesting and followed the original structure of the piece. Another example is the work of researchers at the University of California, Santa Cruz (Mart'inez-Ram'irez et al., 2022), who used ML to create a system that can automatically arrange a melody and chords to create a complete piece of music. This system generated new and unique compositions while maintaining a sense of coherence and structure.

There is also ongoing research in the field of music composition with ML, such as the use of GANs (Figueira and Vaz, 2022) and RNNs (Mansoori and Murali, 2022) to compose music. These methods are still in the early stage of development, and there's room for a lot more research and improvement.

The combination of music arrangement and ML has the potential to revolutionize the way we create, consume, and interact with music. However, this field has challenges and limitations, such as the need for large amounts of data and computational resources, as well as the need to ensure that the generated music is musically pleasing and follows the rules of harmony and structure.

## 8. Music personalization

ML can be used to create music tailored to a particular listener's preferences. Music personalization creates a personalized listening experience for a user based on their preferences, listening history, and other factors. ML can be used to analyze this data and make personalized music recommendations for a user. There are several approaches for music personalization using ML. One approach is collaborative filtering, which utilizes user-item interactions to make personalized recommendations. Another approach is to use content-based filtering, which uses the characteristics of the music itself to make recommendations. Hybrid approaches have also been proposed, combining collaborative and content-based filtering.

One example of an ML music personalisation system is the Pandora Music Genome Project, which combines collaborative and content-based filtering to make personalized music recommendations (Prockup et al., 2015). Another example is Spotify's Discover Weekly feature, which uses ML to create personalized playlists for users based on their listening history and preferences (Ciocca, 2017). However, music personalization also faces challenges such as data privacy and the cold-start problem (new users with no listening history) (Felfernig et al., 2018). Additionally, there is also the challenge of handling user's diverse tastes and the scalability of the system.

In conclusion, music personalization is an area where ML can be applied to improve the user experience. Collaborative filtering, content-based filtering, and hybrid approaches are some of the most common methods used for music personalization. However, some challenges, such as data privacy and scalability, need to be addressed.

### 8.1. Background

In music generation, ML is used to learn the rules and patterns of music and then generate new music based on this learned knowledge. Music generation can be divided into two main categories: symbolic and audio-based. Symbolic music generation uses a set of rules or grammar to generate music, while audio-based generation uses audio samples to generate new music. The most common approach is supervised learning, where an ML model is trained on a music dataset and generates new music based on this training. One of the earliest forms of symbolic music generation was Markov chains, first introduced in the 1960s by Soviet composer and musicologist Andrey Markov. Markov chains are a statistical model that can generate new sequences of symbols based on a given set of rules. In recent years, DL techniques, such as RNNs and LSTM networks, have been used for music generation. These models have been trained on large datasets of MIDI files and audio samples, allowing them to generate new music that is similar in style to the training data. The authors (Dai et al., 2023) presented a novel approach to generating personalized popular music using DL and generative models. A hybrid model that combines the strengths of both imitation learning and structure learning resulted in the creation of musically coherent and appealing pieces of music.

Music generation has explored various techniques such as DL (Hernandez-Olivan and Beltran, 2022), generative models (Hung et al., 2019), and reinforcement learning (RL) (Yang et al., 2017). DL methods, such as RNNs and LSTM networks, have proven to be effective at modeling the sequential nature of music. The authors (Hernandez-Olivan and Beltran, 2022) proposed a DL model that can generate music in the style of a given composer. They trained the model on a dataset of MIDI files from classical composers and showed that it could cause new pieces similar in style to the training data. Generative models, such as VAEs and GANs, have also been used for music generation. In Hung et al. (2019) proposed a VAE-based model to generate new melodies conditioned on a given chord progression. The model was trained on a pop song dataset and could generate new melodies that were similar to the training data. RL has been used to create music by training an agent to make decisions based on a reward signal. Yang et al. (2017) proposed an RL-based model to generate drum patterns in a given style. The model was trained on a dataset of drum patterns and could generate new patterns similar to the training data. The authors (Dai et al., 2023) focused on using DL techniques to generate symbolic music based on the transformer architecture and is trained on a corpus of symbolic music.

There are several approaches have been used to generate a wide range of music, including MIDI sequences (Eck and Schmidhuber, 2002), audio recordings (Engel et al., 2017), and even lyrics (Gao et al., 2022). Various studies and approaches, such as using neural networks to generate music (Hadjeres et al., 2017; Huang et al., 2018), using generative models such as GANs (Creswell et al., 2018) and VAEs (Kingma and Welling, 2013) for music generation (Dong et al., 2017; Yang et al., 2017), and using evolutionary algorithms for music generation (Pachet, 2003). These studies demonstrate the potential of using ML techniques for music generation and the ability to produce high-quality and diverse musical content. However, there are challenges, such as dealing with a lack of data and computational resources and balancing the trade-off between creativity and control in the generated music (Hadjeres et al., 2017; Huang et al., 2018). The authors (Sulun et al., 2022) explored the use of ML techniques for generating symbolic music conditioned on continuous-valued emotions.

In Boulanger-Lewandowski et al. (2012) proposed a method for modeling temporal dependencies in high-dimensional sequences, specifically for polyphonic music generation and transcription. They use a deep neural network architecture to model these dependencies and show that it can generate realisticsounding music. The authors (Schmidhuber, 2015) provided an overview of DL in neural networks, a technique that allows for discovering complex patterns in data through multiple layers of non-linear processing. This technique is used in many of the other papers on music generation. In Creswell et al. (2018) introduced GANs, a method for training DNNs to generate new samples from a given distribution. GANs have been used in music generation to create new pieces of music that are similar to existing ones. Moreover, in Oord et al. (2016) proposed WaveNet, a generative model for raw audio. They use a deep convolutional neural network architecture that can generate new samples of music that sound similar to the training data.

In Graves and Graves (2012) introduced the LSTM network, a recurrent neural network that can model longterm dependencies in sequential data. LSTMs have been used in music generation to model the temporal dependencies in music. The authors (Johnson et al., 2016) proposed Perceptual Losses, a method for training neural networks to generate new samples similar to existing ones in terms of their visual appearance. This technique has been adapted for music generation to create unique pieces of music that are similar to existing ones in terms of their musical properties. Moreover, in Nayebi and Vitelli (2015) proposed a method for music generation using recurrent neural networks. They use a deep LSTM network architecture to model the temporal dependencies in music and show that it can generate new pieces of music that sound similar to the training data. Also, in Benetos and Dixon (2013) proposed Constrained Generative Models for Music Transcription, a method for automatically transcribing music from audio recordings. They use a deep neural network architecture that can model the temporal dependencies in music and show that it can transcribe music with high accuracy. In Pulipati et al. (2021) proposed a method for music genre classification using convolutional neural networks. They use a deep CNN architecture to extract features from music and show that it can classify music into different genres with high accuracy. Furthermore, the authors (Zhang et al., 2016) compared music genre classification using CNN and recurrent neural networks and showed that CNN is more effective in music genre classification.

Overall, the related work on ML for music generation has shown that these techniques can generate a wide range of music, including MIDI sequences, audio recordings, and lyrics. However, many challenges remain to be addressed, such as generating more creative and diverse music and incorporating user feedback into the generation process.

### 8.2. Challenges

Despite the many successes of ML for music generation, several challenges must be overcome. One main challenge is creating unique and diverse music that still follows certain rules and patterns. This requires a deep understanding of music theory and composition and the ability to capture the essence of different music styles. Additionally, using ML for music generation requires large amounts of data, which can be difficult to acquire.

Several challenges need to be addressed in music generation using ML. Some of these challenges include:

**Lack of diversity:** The current models tend to generate music similar to the training data, leading to a lack of diversity in the generated music.
**Difficulty in evaluating the quality of generated music:** It is difficult to evaluate the quality of generated music as it is often subjective.
**Lack of control over the generation process:** The current models do not allow for much control over the generation process, making generating music that meets specific requirements difficult.

### 8.3. Future directions

In the future, ML for music generation will continue to evolve as new techniques and algorithms are developed. One promising area of research is the use of DL, which has the potential to generate music that is even more diverse and unique. Additionally, the use of generative models such as GANs and VAEs will be explored further. Also, using RL to generate music that adapts to the listener's preferences and context is an interesting direction.

There are several future directions that the field of music generation using ML can take.

Some of these directions include:

**Incorporating more diverse training data:** This will help increase the generated music's diversity.
**Developing new evaluation metrics:** This will allow for a more objective evaluation of the quality of generated music.
**Developing more control over the generation process:** This will allow the generation of music that meets specific requirements.

#### 8.3.1. Summary

ML for music generation is a rapidly growing field that has the potential to revolutionize the way we create and experience music. With the use of ML techniques, we can generate new and unique music that follows specific rules and patterns. However, many challenges must be overcome, such as creating diverse and unique music and acquiring large amounts of data. Future research in this field will focus on using DL, generative models and RL to generate music that adapts to the listener's preferences and context. Table 5 illustrates technique strengths and weaknesses, specific goals and requirements for the music generation task.

**Table 5:** Comparison of different ML techniques for music generation.

**Technique**	**Advantages**	**Disadvantages**
RNN	Good at capturing patterns in sequential data and generating new sequences.	Computationally expensive and prone to overfitting, especially with large amounts of data.
GANs	Good at generating new and diverse data, such as musical pieces	Difficult to train, and the results can be unpredictable, with the potential for the generator to produce poor quality outputs.
VAEs	Good at generating high-quality, diverse data in a controllable manner.	Computationally expensive, and the results may not be as diverse as those generated by GANs.
Markov Chain Models	Good at generating sequences that follow a specific pattern or structure, such as musical sequences.	May not generate new and diverse sequences, as they are limited by the data they were trained on.

## 9. Conclusion

In this comprehensive survey, advancements in ML-empowered music were explored, explicitly focusing on four main applications: music recommendation, classification, transcription, and generation. In each areas, the state-of-the-art techniques and models, have highlighted the strengths and limitations. The challenges and trends shaping the future of music processing and analysis using ML has discussed. According to the survey, ML has significantly impacted the music industry by enabling more personalized and sophisticated music recommendations, improved music classification, accurate music transcription, and creative music generation. However, many open research questions still need to be addressed, such as improving the scalability and interpretability of the models, incorporating musical structure and context, and exploring new applications and domains for music analysis. Given the rapidly growing interest in this field, future research will continue to push the boundaries of what is possible with ML in music and lead to even more exciting and innovative applications. As a result, ML will continue to play a critical role in shaping the future of music and its applications.

## Author contributions

JL: Conceptualization, Data curation, Formal analysis, Funding acquisition, Investigation, Methodology, Project administration, Resources, Software, Validation, Visualization, Writing—original draft, Writing—review & editing.
